# A novel methylation signature predicts radiotherapy sensitivity in glioma

**DOI:** 10.1038/s41598-020-77259-9

**Published:** 2020-11-23

**Authors:** Yuemei Feng, Guanzhang Li, Zhongfang Shi, Xu Yan, Zhiliang Wang, Haoyu Jiang, Ye Chen, Renpeng Li, You Zhai, Yuanhao Chang, Wei Zhang, Fang Yuan

**Affiliations:** 1 Department of Pathophysiololgy, Beijing Neurosurgical Institute, Capital Medical University, Beijing, China; 2 Department of Molecular Neuropathology, Beijing Neurosurgical Institute, Capital Medical University, Beijing, China; 3 Department of Neurosurgery, Beijing Tiantan Hospital, Capital Medical University, Beijing, China; 4 Chinese Glioma Genome Atlas Network (CGGA), Beijing, China; 5 Asian Glioma Genome Atlas Network (AGGA), Beijing, China

**Keywords:** Cancer, Genetics, Neuroscience

## Abstract

Glioblastoma (GBM) is the most common and malignant cancer of the central nervous system, and radiotherapy is widely applied in GBM treatment; however, the sensitivity to radiotherapy varies in different patients. To solve this clinical dilemma, a radiosensitivity prediction signature was constructed in the present study based on genomic methylation. In total, 1044 primary GBM samples with clinical and methylation microarray data were involved in this study. LASSO-COX, GSVA, Kaplan–Meier survival curve analysis, and COX regression were performed for the construction and verification of predictive models. The R programming language was used as the main tool for statistical analysis and graphical work. Via the integration analysis of methylation and the survival data of primary GBM, a novel prognostic and radiosensitivity prediction signature was constructed. This signature was found to be stable in prognosis prediction in the TCGA and CGGA databases. The possible mechanism was also explored, and it was found that this signature is closely related to DNA repair functions. Most importantly, this signature could predict whether GBM patients could benefit from radiotherapy. In summary, a radiosensitivity prediction signature for GBM patients based on five methylated probes was constructed, and presents great potential for clinical application.

## Introduction

Glioblastoma (GBM), the most common intracranial malignancy, is a highly therapeutically resistant and fatal disease^1,2^. The median survival time of GBM patients is 14.4 months, and the overall survival (OS) is also limited, varying from less than 3 months to more than 3 years^3,4^. At present, the standard therapy for GBM is to safely resect the tumor to the maximum extent, and then perform chemo- and radiotherapies^5^. However, in clinical practice, it has been found that some GBMs are very susceptible to recurrence due to insensitivity to adjuvant therapy, while others are sensitive to adjuvant therapy. Therefore, the screening of the postoperative adjuvant therapy sensitivity-related biomarkers is of great significance for clinical treatment guidance and prognosis judgment.

DNA methylation, an important genomic epigenetic modification, is of great value in the prediction of cancer treatment sensitivity^6^. The prediction of the sensitivity of GBM patients to chemotherapy (temozolomide) via the MGMT promoter methylation level has been widely conducted in clinical practice^7^. In recent years, the sensitivities to radiotherapy of many tumors, such as those due to esophageal cancer^8^, cervical cancer^9^, laryngocarcinoma^10^, breast cancer^11^, and even GBM^12^, have been found to be related to the alteration of DNA methylation patterns. However, there are no similar prediction models that can predict GBM radiotherapy sensitivity that can be applied in clinical practice. Therefore, the present study was conducted to address this gap.

In this work, a gene methylation signature was constructed to predict the sensitivity of GBM to radiotherapy. First, methylation sites related to radiotherapy sensitivity were screened via the analysis of the methylation microarray data of primary GBM patients with long- and short-term survival after radiotherapy. Subsequently, the most representative methylation probes were screened by LASSO-COX analysis^13^. Finally, five candidate methylation probes were obtained to build a novel methylation signature. The performance of the signature was then validated on the TCGA and CGGA databases, and its related biological functions were explored. It was found that the signature is closely related to the DNA damage repair function, and can be used to predict the prognosis of glioma patients.

## Results

### A novel prognostic and radiosensitivity prediction signature was built based on five methylation probes

**Figure 1:**
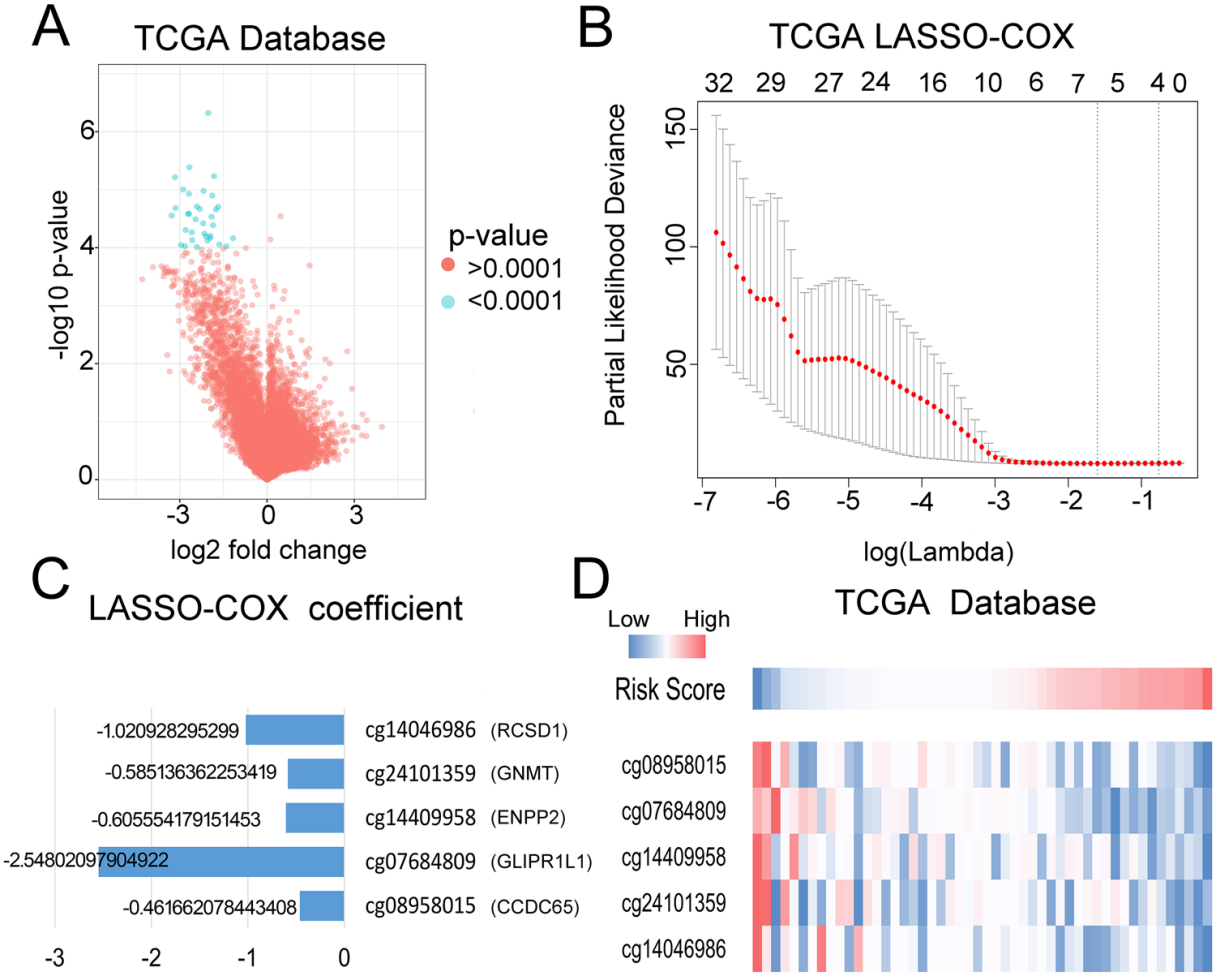
Analysis of methylation using the TCGA database. (**A**) Differentially expressed methylation probes between the radiosensitive and radio-resistant groups. (**B**) Differentially expressed methylation probes determined by LASSO-COX. (**C**) The coefficients of five methylation probes selected by LASSO-COX. (**D**) A novel risk score calculated based on coefficients and the TCGA database.

To determine the methylation probes associated with radiotherapy sensitivity, 50 primary GBM patients who underwent radiotherapy only after surgery were assigned into either the radiosensitive group or radio-resistant group. A t-test was performed to compare the differentially expressed methylation probes between the two groups, and 32 probes were obtained for further analysis (Fig. 1A). To further screen out the most representative methylation probes, five probes and their corresponding coefficients were identified by LASSO-COX dimension reduction analysis (Fig. 1B,C). Finally, a novel prognostic and radiosensitivity prediction signature was constructed based on the probes (Fig. 1D). The in-depth analysis of this prediction signature in gliomas was conducted as follows.

### The landscape of signature and clinical characters in gliomas

**Figure 2:**
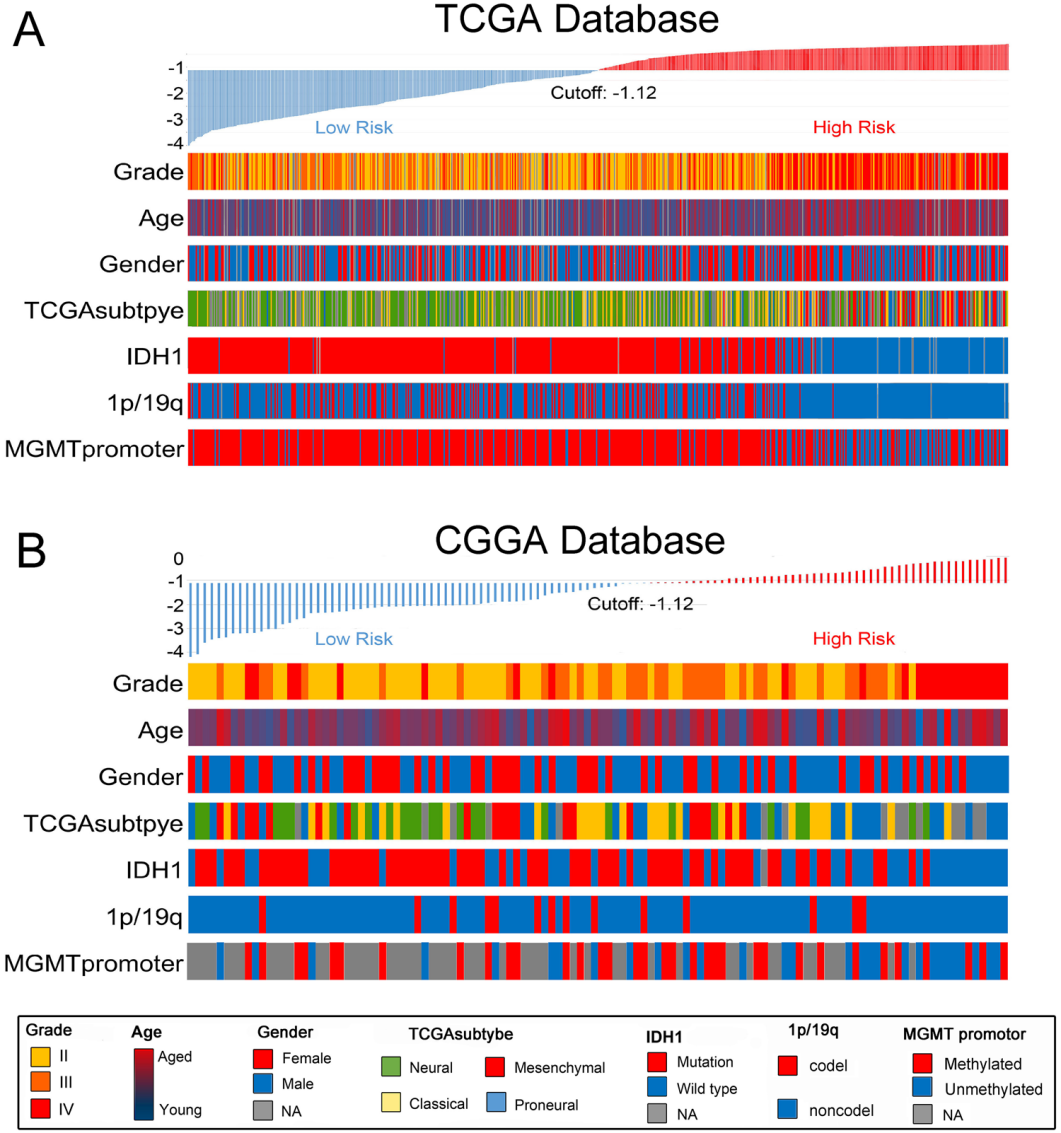
The relationships between risk score and clinical information (**A**) in the TCGA database and (**B**) in the CGGA database.

The prediction signature was applied to the TCGA database to further validate its predictive performance. The risk scores of 928 patients in the TCGA database were calculated based on the expressions of their representative methylation probes, and the median of the score was defined as the cutoff. A heatmap revealed the relationships between risk scores and WHO grade, age, gender, TCGA subtype, IDH1 status, 1p/19q status, and MGMT promoter (Fig. 2A), and it was determined that, excluding gender, each characteristic had an asymmetrical distribution. Higher-grade, older-aged, IDH1-wild, 1p/19q-non-codeletion, and unmethylated-MGMT promoter patients were mostly distributed in the higher-risk segment. The same method was used to investigate the CGGA database, and 116 patients were included (Fig. 2B). The cutoff of the risk group in the CGGA database was defined as − 1.12, which was the same as that of the TCGA database. It is clear that the relationships between the risk scores and WHO grade and IDH1 status in the CGGA database were the same as those in the TCGA database. The statistical analysis of these relationships was subsequently carried out.

### The relationship between signature and clinical characters in gliomas

**Figure 3:**
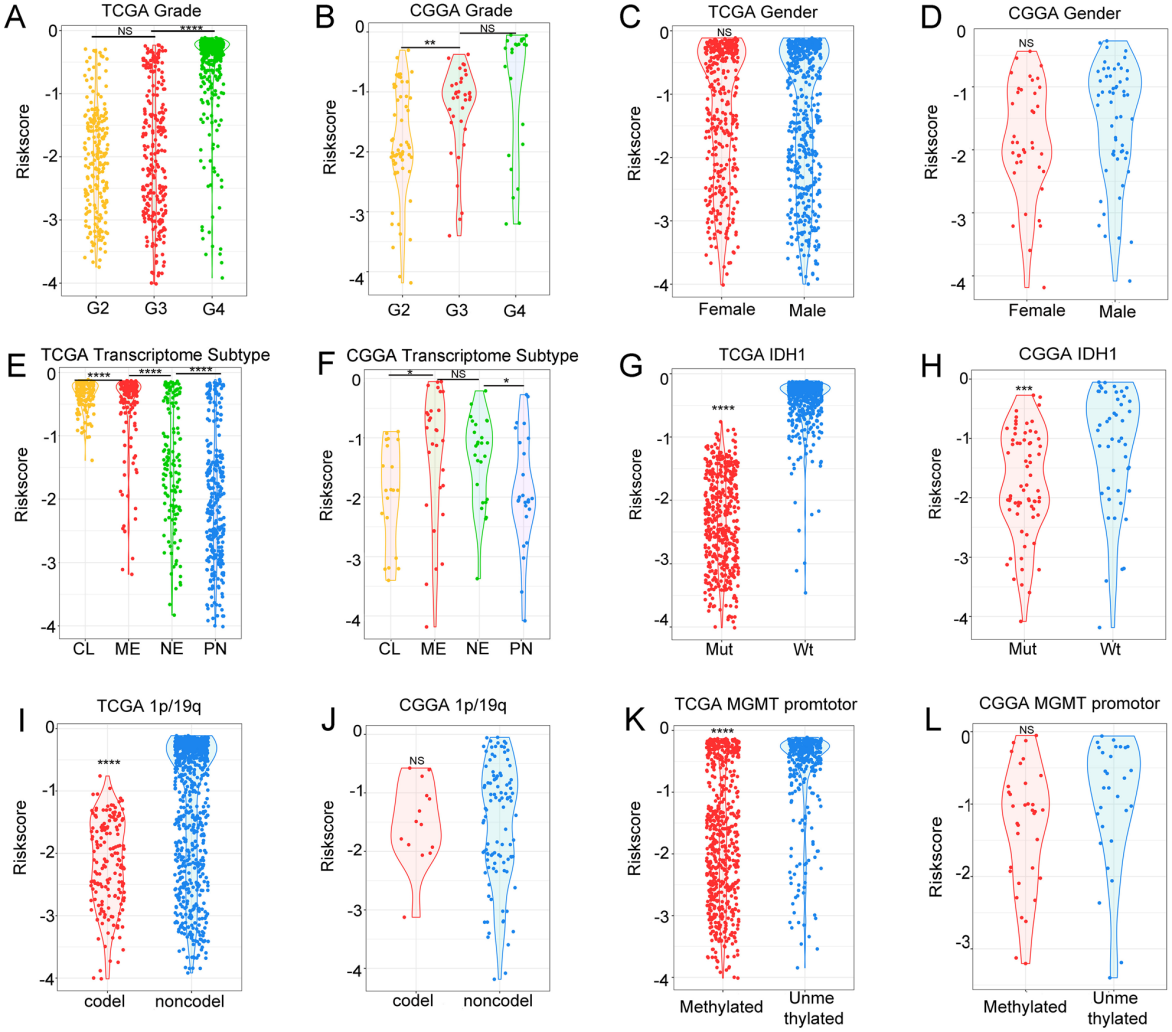
Correlations between risk scores and clinical features in the TCGA and CGGA databases. (**A**,**B**) Correlation between risk score and WHO grade in the TCGA and CGGA databases. (**C**,**D**) Correlation between risk score and gender in the TCGA and CGGA databases (*CL* classical, *ME* mesenchymal, *NE* neural, *PN* proneural). (**E**,**F**) Correlation between risk score and transcriptome subtype in the TCGA and CGGA databases. (**G**,**H**) Correlation between risk score and IDH1 status in the TCGA and CGGA databases. (**I**,**J**) Correlation between risk score and 1p/19q status in the TCGA and CGGA databases. (**K**,**L**) Correlation between risk score and MGMT promoter status in the TCGA and CGGA databases.

The relationships between the signature risk score and various clinical features were investigated via the TCGA and CGGA databases. In the TCGA database, it was found that the risk score in WHO IV gliomas was higher than those in WHO II and WHO III gliomas (Fig. 3A), and the same trend was found in the CGGA database (Fig. 3B). In both the TCGA and CGGA databases, the risk scores between different genders were found to have no significant differences (Fig. 3C,D). Among the transcriptome subtypes, the risk score was found to be relatively higher in the mesenchymal subtype (Fig. 3E,F). In terms of molecular pathology, the risk score was found to be much higher in IDH1 wild-type and 1p/19q-non-codeletion gliomas (Fig. 3G,H). The MGMT promoter methylation status was found to be an important indicator of chemosensitivity in gliomas, and the correlations between the risk score and MGMT promoter methylation status suggests that the former might be related to chemotherapeutic drug sensitivity (Fig. 3I–L). Some difference analysis of the CGGA database revealed the same trends as those of the TCGA database but were not statistically significant, which was most likely due to insufficient samples.

### The signature was closely related to DNA repair functions

**Figure 4:**
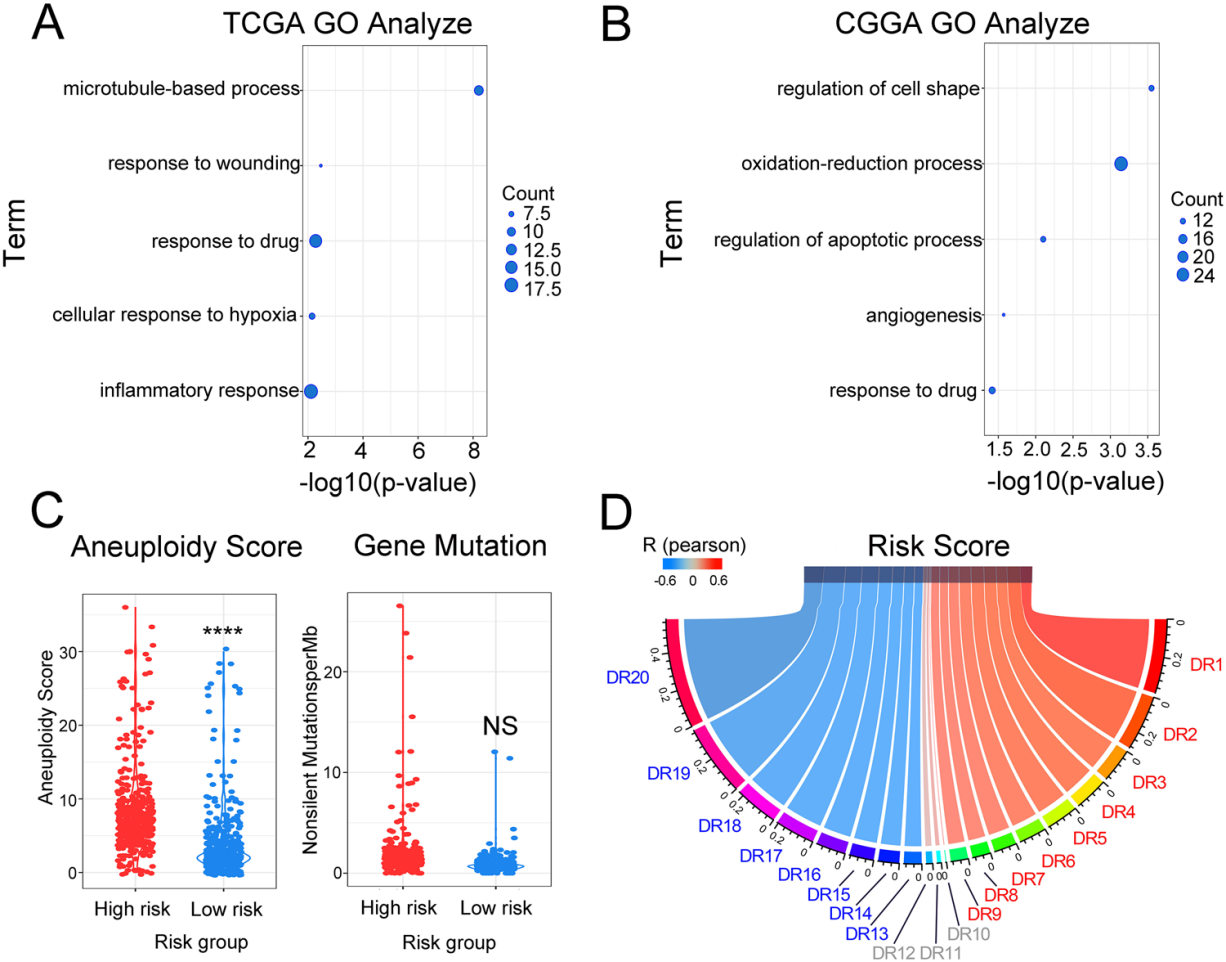
The functions analyses of the signature. (**A**,**B**) GO function enrichment analysis in the TCGA and CGGA databases. (**C**) Correlation between the risk group and aneuploidy score or gene mutation in the TCGA database. (**D**) Correlation between the risk score and DNA repair functions in the TCGA database. (DR1: Homologous recombination; DR2: Repair of DNA-topoisomerase crosslinks; DR3: Fanconi anemia/tolerance and repair of DNA crosslinks and other adducts in DNA; DR4: Editing and processing nucleases; DR5: Modulation of nucleotide pools; DR6: Base excision repair (BER)/DNA glycosylases: major altered base released; DR7: Chromatin structure and modification; DR8: Other BER and strand break joining factors/Other BER and strand break joining factors; DR9: Nucleotide excision repair (NER)/(XP = xeroderma pigmentosum); DR10: Direct reversal of damage; DR11: Ubiquitination and modification; DR12: Mismatch excision repair (MMR); DR13: Other identified genes with known or suspected DNA repair function; DR14: Genes defective in diseases associated with sensitivity to DNA damaging agents; DR15: Other conserved DNA damage response genes; DR16: TFIIH/Catalyzes unwinding in pre-incision complex; DR17: Non-homologous end-joining; DR18: DNA polymerases (catalytic subunits); DR19: Poly(ADP-ribose) polymerase (PARP) enzymes that bind to DNA; DR20: NER-related).

Gene ontology (GO) function enrichment analysis was performed to explore the biological functions associated with the signature. The five biological functions in the TCGA database were the “microtubule-based process,” “response to wounding,” “response to drug,” “cellular response to hypoxia,” and “inflammatory response” (Fig. 4A). In CGGA, the most risk score-related biological functions were found to be “regulation of cell shape,” “oxidation–reduction process,” “regulation of apoptotic process,” “angiogenesis,” and “response to drug” (Fig. 4B). To integrate the results, “cell damage repair” and “response to drug” were highlighted, and it was concluded that the signature was associated with DNA mutation and the DNA repair functions in gliomas. As was expected, patients in the high-risk group had much higher aneuploidy scores and more gene mutation, which might have been due to the changed DNA repair functions (Fig. 4C). To verify this hypothesis, the correlations between the radiosensitivity prediction signature and DNA repair signatures obtained from the MD Anderson Cancer Center were investigated. Impressively, the radiosensitivity prediction signature was found to be significantly correlated with almost all DNA repair functions (Fig. 4D). In summary, the radiosensitivity prediction signature risk score was found to be likely to be associated with genomic instability caused by DNA repair function changes, which are one of the causes of poor prognosis.

### The radiosensitivity prediction signature, as an independent prognostic factor, is a predictor of radiosensitivity in glioma patients

**Figure 5:**
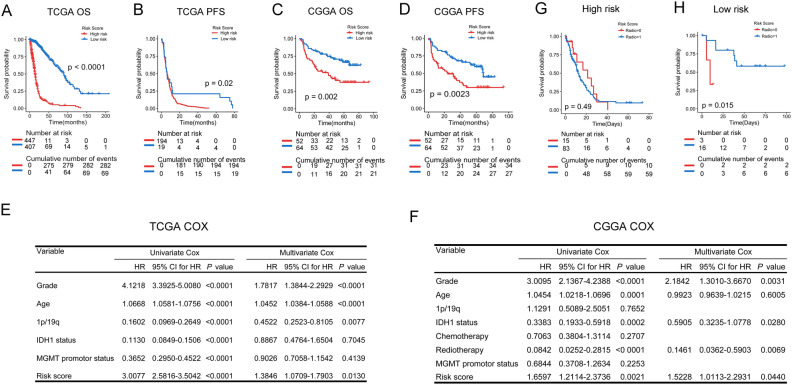
The signature was found to well predict the survival information. (**A**,**B**) Risk score replied to survival information in the TCGA database (OS: *P* < 0.0001, PFS: *P* = 0.02). (**C**,**D**) Risk score replied to survival information in the CGGA database (OS: *P* = 0.002, PFS: *P* = 0.0023). (**E**,**F**) Univariate and multivariate Cox regression in the TCGA and CGGA databases. (**G**,**H**) Correlation between high or low risk score and the prognostic analysis after combining the patients in the CGGA and TCGA databases (High-risk: *P* = 0.49, Low-risk: *P* = 0.015).

**Figure 6:**
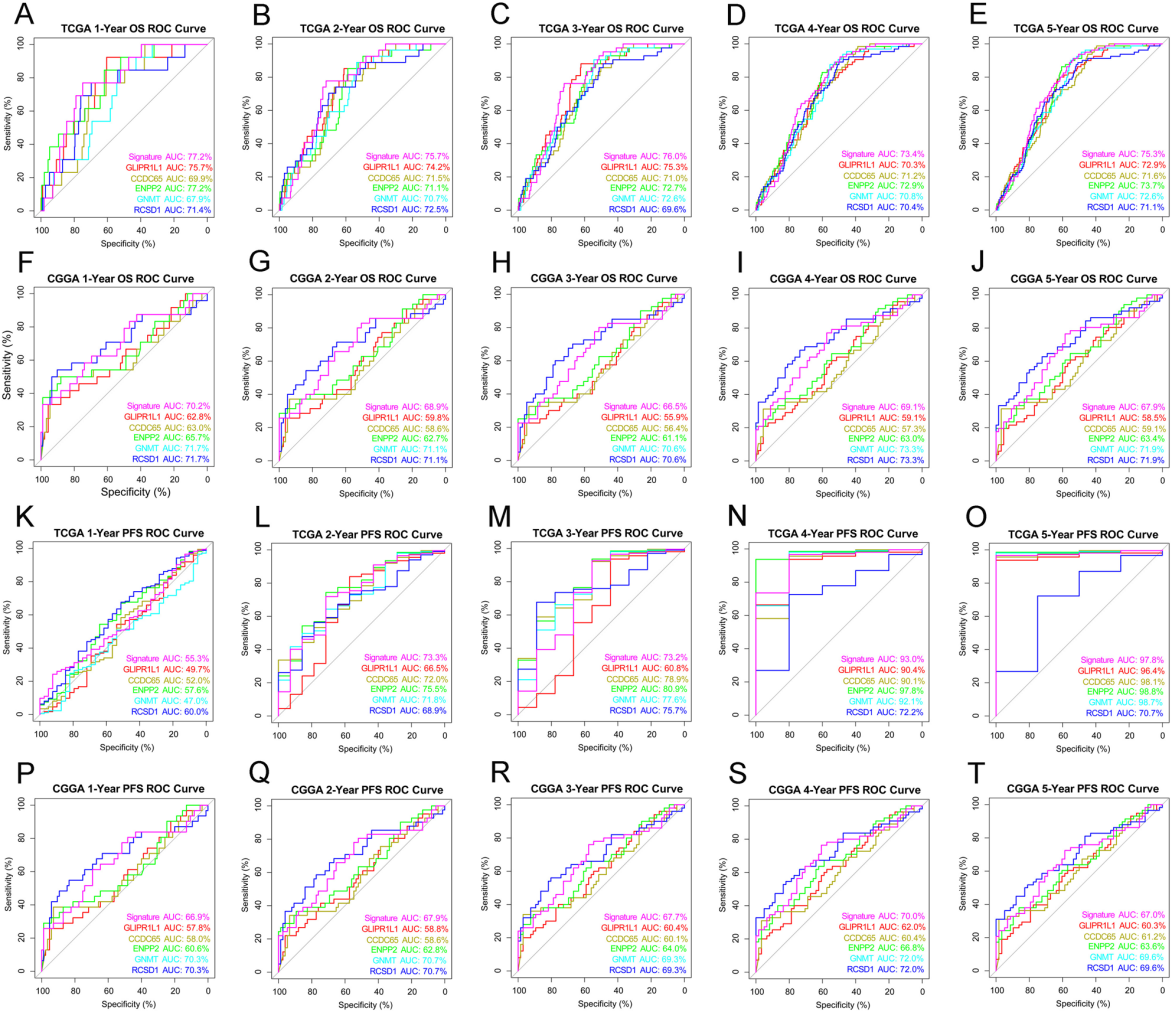
ROC curves showed the predictive efficiency of the signature and the 5 probes respectively on 1,2,3,4 and 5 years of OS or PFS in TCGA and CGGA databases. (The curve of GNMT was covered by the curve of RCSD1 for coinciding in CGGA database) (A-T).

The signature was applied to the TCGA and CGGA databases to evaluate its performance of prognostic prediction. As shown in Fig. 5A–D, patients in the high-risk group had a shorter OS and PFS than low-risk patients. In addition, univariate and multivariate COX regressions were performed to evaluate the comprehensive prognostic prediction value of the risk score and clinicopathologic characteristics. Univariate COX regression revealed that the WHO grade, age, 1p/19q status, IDH1 status, MGMT promoter status, and signature risk score were notably related to the survival in the TCGA database (*P* < 0.0001). Additionally, the multivariate COX regression revealed that the WHO grade, age, 1p/19q status, and risk score were screened as independent prognostic factors (Fig. 5E). In the CGGA database, the WHO grade, IDH1 status, radiotherapy, and risk score were found to be independent prognostic factors (Fig. 5F). By applying the risk score to the subgroups of grade, IDH1 status, 1p19q status, and MGMT methylation status, reliable predictions were obtained (Figs. S2, S3). Due to the limitations of patient numbers, the patients from the CGGA and TCGA databases were combined for further analyses. In both databases, 117 patients who only underwent postoperative radiotherapy were found. According to the radiosensitivity prediction signature, 98 patients were classified into the high-risk group, and 19 patients were classified into the low-risk group. Prognostic analysis demonstrated that patients in the low-risk group, rather than in the high-risk group, significantly benefitted from postoperative radiotherapy (Fig. 5G,H). The receiver operating characteristic (ROC) curve analysis showed that risk score had a good efficiency for predicting the 1–5 years of OS and PFS of patients TCGA and CGGA databases (Fig. 6).

## Discussion

Many studies have shown that high tumor heterogeneity is an important feature of GBM, and leads to significant differences in therapeutic effect^14^. The development of genome sequencing technology has provided substantial assistance with tumor heterogeneity research^15^. In recent years, many studies have focused on the treatment sensitivity of glioma patients based on transcriptome sequencing. However, due to the highly variable nature of the transcriptome, it was difficult to apply the results of these studies to clinical treatment guidance. Currently, emerging evidence has been found to confirm epigenetics; particularly, DNA methylation affects the genesis and development of other solid tumors, and reflects sensitivity to radiotherapy and chemotherapy^16^. Additionally, the methylation level of the MGMT promoter region has been widely used as a predictor of chemotherapeutic drug sensitivity in gliomas. To find a stable predictor for the sensitivity of radiotherapy, an important adjuvant therapy for gliomas, the methylation data of the TCGA and CGGA databases was mined in this study. According to previous reports, patients with a survival time of greater than 18 months after postoperative radiotherapy were defined as long-term survivors (radiotherapy-sensitive group), and those with a survival time of less than 9 months were defined as short-term survivors (radiotherapy-insensitive group)^17^. By comparing the gene methylation differences and dimensionality analysis of the two groups, five candidate methylation probes were ultimately obtained.

These five methylation probes were located in the promoter regions (cg08958015, cg14046986, and cg24101359 were located in the CpG island, and cg14409858 and cg07684809 were located in the non-CpG island) of five different genes, namely *CCDC65*, *RCSD1*, *GNMT*, *ENPP2*, and *GLIPR1L1*. Gene regulation sites regulate gene expression, and thus play a role in DNA methylation. All five genes have negative correlations with the corresponding DNA methylation level, suggesting that the methylation of these five loci could affect different biological functions by affecting the expressions of the genes. To explore the mechanism of the risk score in prediction, the biological functions of these five genes were reviewed. *CCDC65* is an important subunit of the nexin dynein regulatory complex (N-DRC), which is important in the regulation of ciliary and flagellar motility^18^. Additionally, other researchers have found that cilium can combine with zinc finger E-box binding homeobox 1 (ZEB1), leading to chemoresistance and radioresistance in GBM^19,20^. Therefore, *CCDC65* is speculated to impact radiosensitivity via cilium and ZEB1. The *RCSD1* gene encodes the phosphoprotein CapZ-interacting protein (CapZIP), which contributes to the regulation of actin filament assembly and plays an important role in actin filament binding, which is an important biological behavior of tumors^21^. *GLIPR1L1* participates in the encoding of the glioma pathogenesis-related protein (GLIPR1), a member of the CAP superfamily, and could promote the proliferation, survival, and invasion of glioma cells and inhibit apoptosis^22,23^. *ENPP2* encodes ectonucleotide pyrophosphatase/phosphodiesterase 2(ENPP2), also known as Autotaxin, and catalyzes the generation of the lipid-signaling molecule lysophosphatidic acid (LPA)^24^, which participates in the development of the nervous system and tumor progression^25,26^. Finally, *GNMT* plays a role in hepatocarcinogenesis^27^. Thus, these five genes are associated with the malignant progression and treatment resistance of tumors.

**Figure 7:**
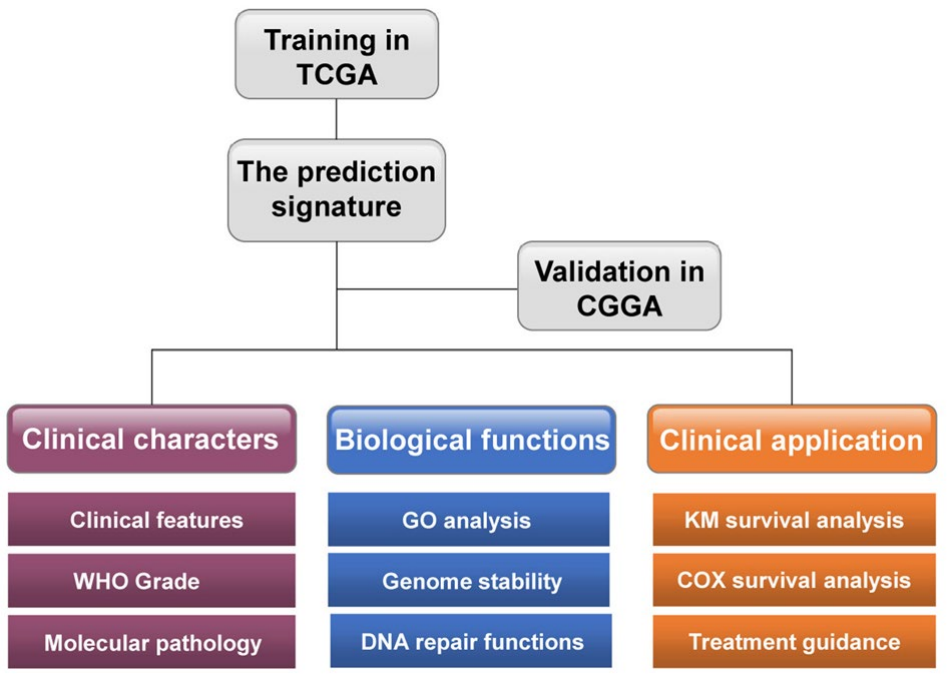
The workflow.

Finally, a novel gene signature was established based on these five genes. To facilitate the clinical application of this signature, the cutoff values of the risk groups were the median values of the risk scores of the experimental groups (15), and the fixed cutoff value was − 1.12. And as the validation, the CGGA database employed the same cutoff value to TCGA database (Fig. 7, S1)^28,29^. The relationships between the signature risk score and the clinicopathologic and molecular features of gliomas in the TCGA database were analyzed. The results revealed that the risk score has a significant correlation with the clinical features of glioma, including TCGA transcriptome subtypes, WHO grade, IDH1 status, 1p/19q status, and MGMT promoter methylation status. These relationships were also verified in the CGGA database. The biological functions associated with the risk score were also analyzed, and DNA repair functions and genomic instability were highlighted. As genomic alteration has been found to be an important feature of gliomas with poor prognosis, it was believed that the risk score could predict the prognosis of glioma patients by reflecting differences in DNA repair status after radiotherapy. Furthermore, the differences in DNA repair functions were also likely to be the internal cause of the differences in the radiotherapy sensitivity of glioma patients. This is why risk score, which is closely related to DNA repair functions, can reflect the radiotherapy sensitivity of glioma patients. Subsequent prognostic analysis also confirmed that not only is the risk score an independent prognostic factor, but it could also predict postoperative radiotherapy sensitivity in glioma patients.

In conclusion, a stable prognostic prediction model based on methylation probe databases was constructed. On the macroscopic scale, the relationships between risk score and glioma clinical features and molecular pathology were revealed. On the microcosmic scale, it was found that the risk score is associated with DNA repair functions and genomic instability in gliomas. In terms of clinical transformation, the retrospective studies revealed that the risk score could predict the prognosis and radiosensitivity of glioma patients. These three aspects provide possible solutions to the clinical problem of judging the treatment effect of gliomas.

## Methods

### Patient cases and expression data processing

This study involved data from 1044 cases of glioma patients sourced from the CGGA and TCGA databases, and the clinical information is listed in Table 1. Among all the samples, there were 50 primary glioblastoma cases from the TCGA database that met the prognosis and treatment selection criteria. All methylation values used were beta values. All the TCGA expression data and survival information were downloaded from the official TCGA website (https://cancergenome.nih.gov). Additionally, 116 cases from the CGGA database were included in this study. Each case was diagnosed by two neuropathologists based on the 2007 WHO classification guidelines. Tumor samples were acquired from newly excised tissue. The OS and progression-free survival (PFS) were calculated from the date of diagnosis to the end of the follow-up investigation. Sample collection and data analysis were approved by Beijing Tiantan Hospital institutional review board (IRB) and wrote informed consent was obtained from each participate. The study was conducted in accordance with the European Good Clinical Practice requirements (Declaration of Helsinki). The informed consents were obtained from all subjects.

**Table 1:** Clinical information of patients.

Characteristic	No. of patients (TCGA)	No. of patients (CGGA)
**Age at diagnosis**		
Mean	50.4	39.2
Standard deviation	15.9	11.5
**Gender**		
Male	492	67
Female	366	49
Not available	70	0
**Grade**		
WHO II	215	57
WHO III	240	35
WHO IV	403	24
Not available	70	
**IDH1 mutation**		
Mutation	450	68
Wildtype	426	47
Not available	52	1
**1p/19q codeletion status**		
Codeletion	170	14
Non-codeletion	743	102
Not available	15	0
**Radiotherapy**		
Yes	341	65
No	22	5
Not available	565	46
**Chemotherapy**		
Yes	273	40
No	90	30
Not available	565	46

### Candidate methylation probe selection and methylation signature building

Data from the TCGA database was identified as the experimental group, and that from the CGGA database was identified as the verification group. According to previous research, an OS time of less than 9 months was defined as short survival, and that more than 18 months was defined as long survival^17^. Student’s *t*-test was employed to analyze the data from the TCGA database to find radiotherapy sensitivity-related methylation probes, and included data from 43 short-survival and 7 long-survival primary GBM patients who underwent postoperative radiotherapy. Thirty-two probes that were significantly associated with radiotherapy resistance or sensitivity (*P* < 0.0001) were obtained for subsequent analysis. LASSO-COX analysis was then carried out for these candidate probes, and five candidate methylation probes were ultimately obtained. The risk score of each patient was calculated by the sum of the corresponding methylation probe values multiplied by the LASSO-COX coefficient.

### Biological functional enrichment scores

The biological functional enrichment score of each patient was generated via gene set variation analysis (GSVA) based on tumor transcriptome sequencing data. GSVA was performed using the default parameters of the GSVA package in R as described in a previous study^30^. The gene list for each biological function was downloaded from the AmiGO2 web portal (https://amigo.geneontology.org).

### LASSO-COX dimension reduction analysis

LASSO-COX dimension reduction analysis was performed via the glmnet and survival packages in R. Finally, 5 genes and corresponding lambda values (CCDC65: − 0.461662078443408, GLIPR1L1: − 2.54802097904922, ENPP2: − 0.605554179151453, GNMT: -0.585136362253419, and RCSD1: − 1.020928295299) were obtained. The risk score of each patient was the sum of the DNA methylation degrees and their corresponding lambda values.

### Statistical analyses

All statistical analyses were conducted using the R programming language (https://www.r-project.org/, v3.5.0), SPSS 25.0 software, GraphPad Prism 7 software (GraphPad Software, Inc., La Jolla, CA), and the DAVID website (https://david.ncifcrf.gov/summary.jsp). The prognostic significance was assessed by Kaplan–Meier curves. Gene ontology (GO) analysis were carried out to illustrate the signature survival differences observed between high- and low-risk score groups. The correlations between two variables were verified by Pearson’s correlation analysis, and P < 0.05 was regarded as statistically significantly different.

## Conclusions

Overall, a novel signature was established to predict the prognosis and radiosensitivity of glioma patients, which can provide possible solutions to the clinical problem of glioma treatment.

## Supplementary information

Supplementary Legends.

Supplementary Figure S1.

Supplementary Figure S2.

Supplementary Figure S3.

## Data Availability

The TCGA and CGGA data used to support the findings of this study were sourced from https://cancergenome.nih.gov/ and www.cgga.org.cn/, respectively.
