# Supplementary material for: A novel methylation signature predicts radiotherapy sensitivity in glioma
Source: Sci Rep. 2020 Nov 23;10:20406. doi: 10.1038/s41598-020-77259-9 (PMC7683673; doi:10.1038/s41598-020-77259-9)
Supplement: Supplementary file 1 — Supplementary Legends. [file 41598_2020_77259_MOESM1_ESM.docx]

**Fig. S1.** The modeling process

**Fig. S2.** TCGA database.

**Fig. S3.** CGGA database.
