# Supplementary figures and images for: A novel methylation signature predicts radiotherapy sensitivity in glioma
Source: Sci Rep. 2020 Nov 23;10:20406. doi: 10.1038/s41598-020-77259-9 (PMC7683673; doi:10.1038/s41598-020-77259-9)

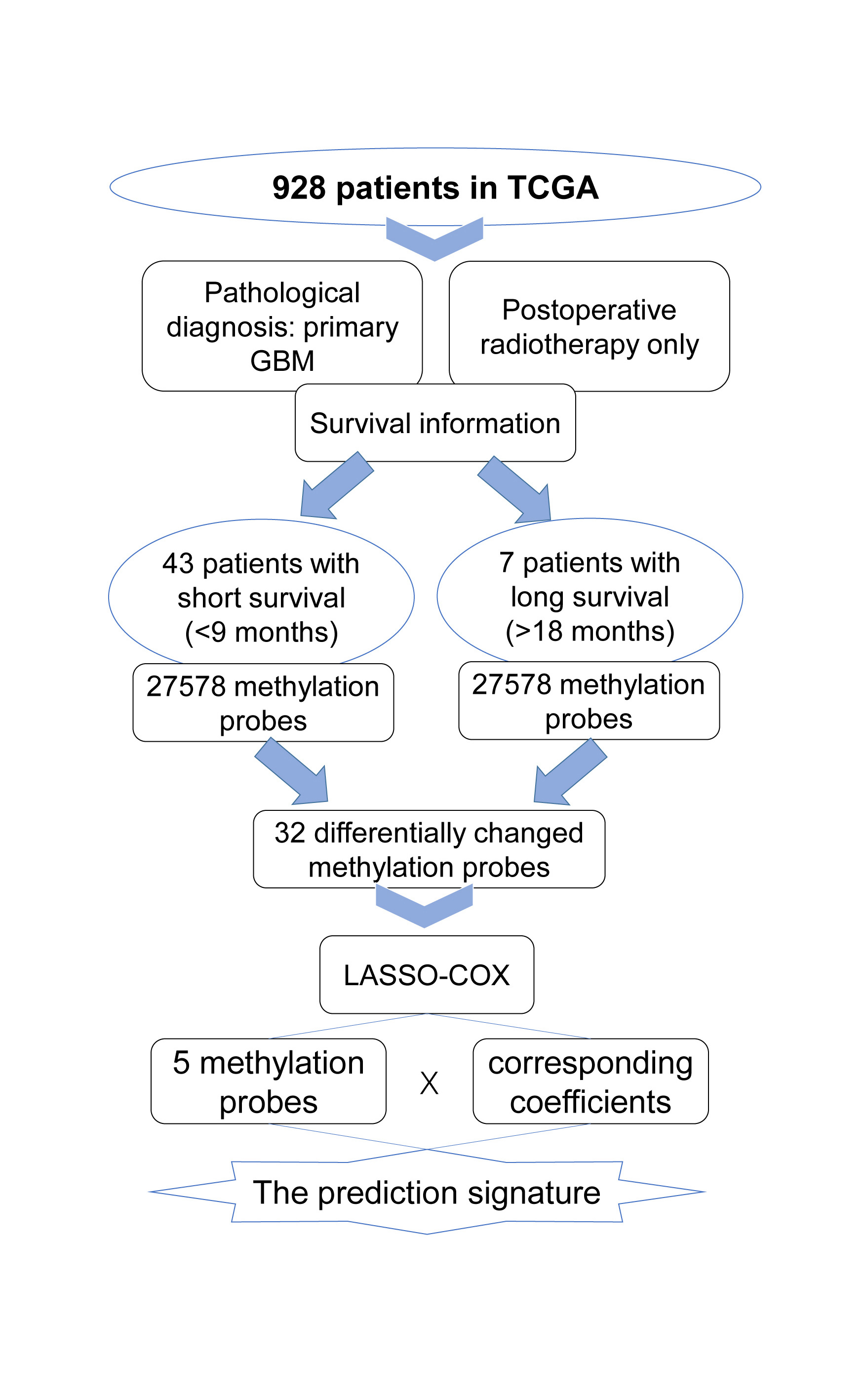

Supplement: Supplementary file 2 — Supplementary Figure S1. [file 41598_2020_77259_MOESM2_ESM.jpg]

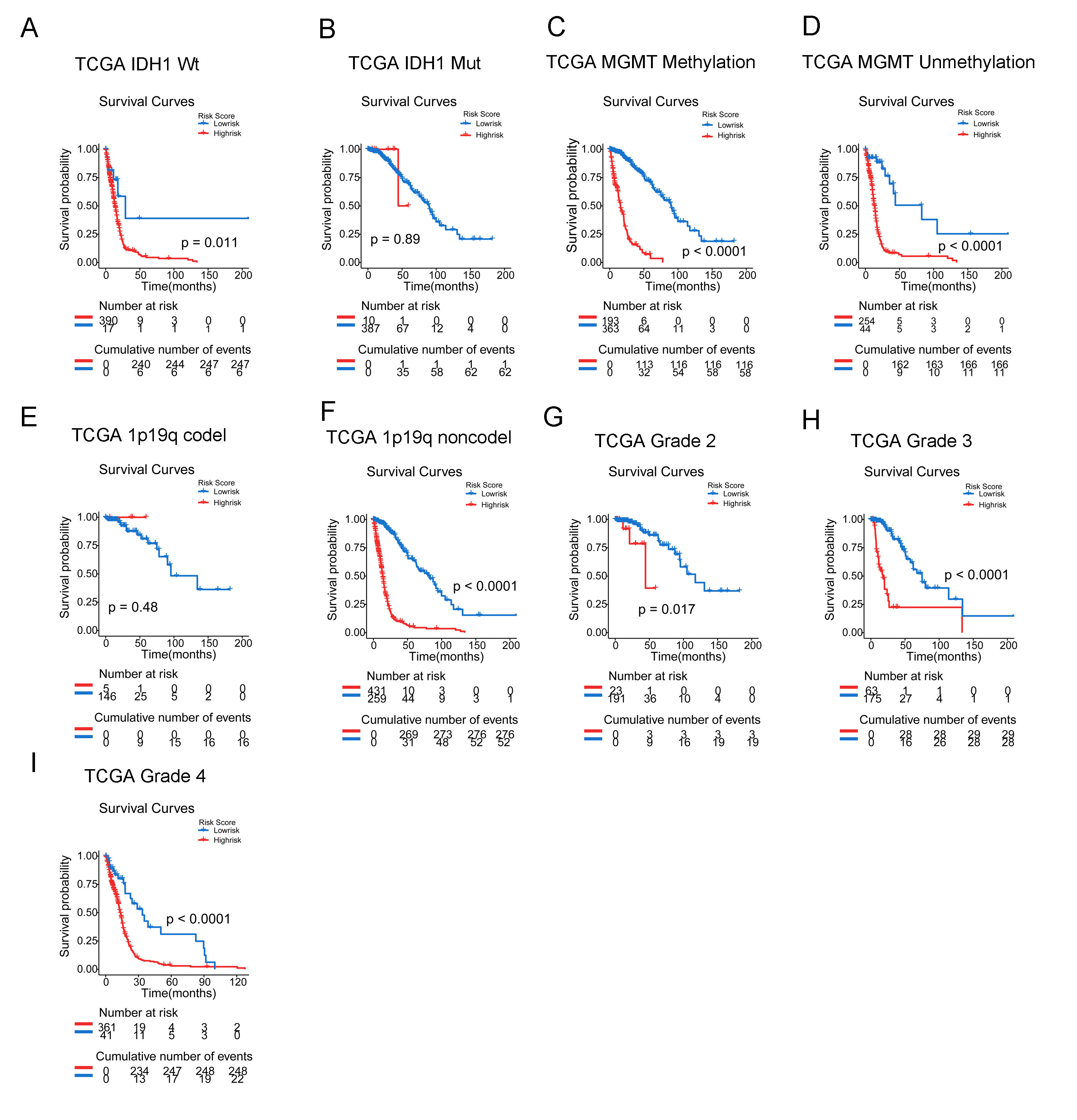

Supplement: Supplementary file 3 — Supplementary Figure S2. [file 41598_2020_77259_MOESM3_ESM.jpg]

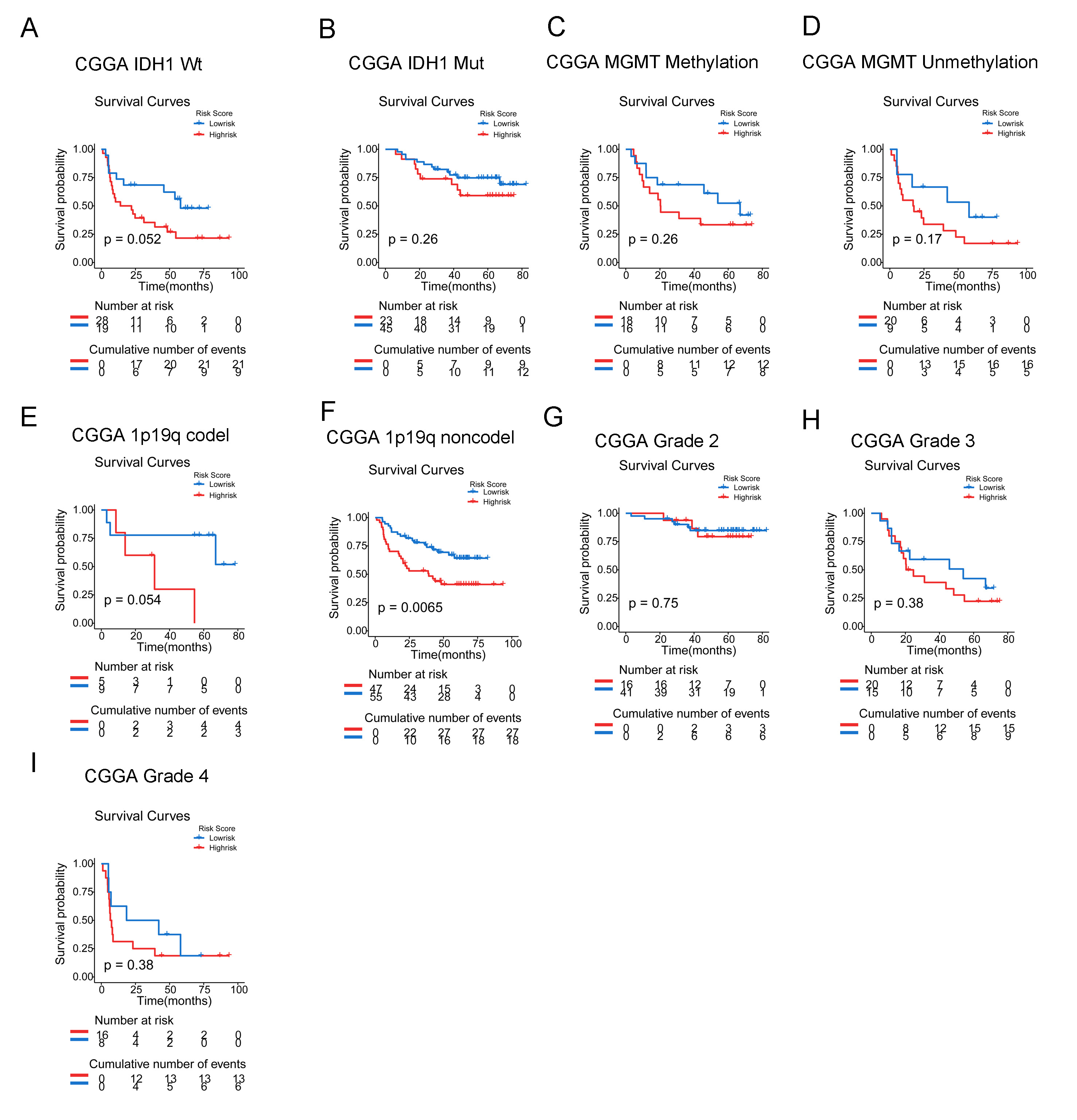

Supplement: Supplementary file 4 — Supplementary Figure S3. [file 41598_2020_77259_MOESM4_ESM.jpg]
